# Prevalence of and attitudes towards complementary therapy use for weight after breast cancer in Australia: a national survey

**DOI:** 10.1186/s12906-019-2747-6

**Published:** 2019-11-21

**Authors:** Carolyn Ee, Adele Elizabeth Cave, Dhevaksha Naidoo, John Boyages

**Affiliations:** 1 NICM Health Research Institute, Western Sydney University, Locked Bag 1797, Penrith, NSW 2751 Australia; 2 Department of Clinical Medicine, Faculty of Medicine and Health Science, Macquarie University, Sydney, NSW 2109 Australia

**Keywords:** Breast cancer, DCIS, Complementary medicine, Overweight, Obesity, Weight gain, Australian women, National survey, Prevalence

## Abstract

**Background:**

Weight gain is common after breast cancer (BC) treatment and may increase the risk of disease recurrence. Complementary medicine (CM) use is high amongst BC patients. This paper describes the use of CM from a cross-sectional self-administered survey on prevalence and management of weight after BC.

**Methods:**

Use of CM was assessed using a question modified from the I-CAM Questionnaire. Participants were asked to rate perceived effectiveness, advantages and disadvantages, and which CM they were willing to use for weight management if there was evidence for effectiveness. The survey was emailed to members of the Breast Cancer Network Australia Survey and Review Group, the largest consumer advocacy group in Australia for people with breast cancer.

**Results:**

There were a total of 309 responses. Three quarters had used CM in the past 12 months. One third had tried CM for weight loss. Yoga, meditation and pilates were perceived to be effective for weight loss. Perceived advantages of CMs for weight loss were the ability to improve general wellbeing, relaxation, and being non-pharmacological while disadvantages were financial cost, finding a reliable practitioner, and lack of research for effectiveness. Three quarters would be willing to try CM for weight loss if there was evidence for effectiveness, with the most popular CMs being acupuncture, relaxation, yoga, supplements, and meditation.

**Conclusions:**

The high use of CM in this group is consistent with previous research. Our research suggests that BC survivors would use acupuncture, meditation, supplements and yoga for weight loss if supported by scientifically-credible evidence. Research into the effectiveness of these treatments on weight loss after BC is warranted.

## Background

Complementary medicine (CM) or complementary therapies refer to ‘a group of diverse medical and healthcare systems, practices and products that are not generally considered part of conventional medicine’ [1]. In this paper, the terms “complementary medicine”, “complementary therapies” and “complementary and alternative medicine” are used synonymously. Boundaries within CM and between the CM domain and that of the dominant system are not always clearly defined or fixed [2]. Women with breast cancer (BC) are the most likely group to use CM out of all cancer patients [3] with CM use reportedly as high as 75% [4]. Patients with breast cancer using CM are mostly younger and more highly educated than non-CM users in the majority of studies. Some studies have also shown that women with breast cancer using CM had higher income than those who did not [3]. An association between CM use and more advanced breast cancer at diagnosis has also been found [5].

Weight gain is common after a breast cancer diagnosis and may increase the risk of disease recurrence and all-cause mortality, increase the burden of chronic disease from obesity-related disorders such as cardiovascular disease and diabetes, and have a significantly negative impact on quality of life [6]. Weight gain after a BC diagnosis is thought to be multifactorial and related to the use of systemic treatment as well as changes in lifestyle [6, 7]. There is emerging evidence for the use of some CM to assist weight loss in the general population. However, little is known about the use of CM for weight loss amongst women with BC, making it important to understand patterns and drivers of use of CM amongst women with BC.

The aim of this national survey was to describe the use of CM for weight management after BC in Australian women.

## Methods

### Study design and inclusion criteria

We conducted a cross-sectional self-administered anonymous survey using Qualtrics online survey software [8]. Any woman living in Australia who self-identified as having BC was eligible to complete the survey. We recruited mainly from the Breast Cancer Network Australia (BCNA) Review and Survey Group (*n* = 1857), representing approximately 2% of all BCNA members, who had agreed to receive emails about research studies. BCNA is the largest breast cancer advocacy group in Australia, and their Review and Survey Group is one of the largest breast cancer consumer groups available for research, representing an important source of feedback for the research community. By limiting research at BCNA to the Review and Survey group, researchers have access to women who are engaged in the research process, while the remainder of BCNA members are protected from frequent research requests. We also recruited through other online communities (women’s health organization social media pages and online breast cancer support groups in Australia).

The survey was emailed on December 5th, 2017 and a reminder email was sent to 1835 members on January 15th, 2018 (Appendix). Ethics approval was provided by the Human Research Ethics Committee, Western Sydney University (H12444, Oct 2017).

### Survey instrument

This study is drawn from a larger study exploring the prevalence, predictors and management of weight after breast cancer amongst women in Australia (Ee C, Cave A, Naidoo D, Boyages J. Weight before and after a diagnosis of breast cancer or Ductal Carcinoma In Situ: a national Australian survey/under review). The survey was developed after reviewing previous literature on weight after breast cancer and was later revised to include feedback from six BCNA representatives and several health researchers. The 60-item survey included questions on the sociodemographic characteristics, medical details such as diagnosis and treatment, lifestyle habits, and weight and weight management of women (see Appendix for further details). In this paper, we report on complementary therapy use for any condition and for weight management. Further analyses from our data will include predictors of weight gain in our sample, but are not reported in this manuscript.

### Weight after diagnosis

Women were asked to self-report their weight in kilograms (kg) at the time of diagnosis, and current weight and height (in meters). Body Mass Index (BMI) was calculated from weight and height as weight/height^2^ and reported in kg/m^2^. BMI was classified as underweight (< 20 kg), healthy weight (BMI ≥ 20 and < 25), overweight (BMI ≥ 25 and < 30) and obese (BMI ≥ 30) [9]. The pattern of weight since diagnosis was also assessed as “gained weight overall”, “lost weight overall”, “weight stable” or “weight has fluctuated a great deal”. Weight at diagnosis was reported by 90% of total respondents (277 women) and current weight by 95% of respondents (293 women).

### Complementary therapies

Women were asked about their use of CM for any condition, and also specifically for weight loss, in the past 12 months. This question was modified from the International Questionnaire to Measure.

Use of Complementary and Alternative Medicine (I-CAM Questionnaire) [10] which is culture-neutral and does not promote one modality over another although we have slightly modified the terminology to suit an Australian population [10]. CM use is divided into visits to complementary therapists (e.g. acupuncturists, chiropractors), use of herbal medicine and dietary supplements (defined as vitamins, minerals, herbs and antioxidants), and of self-help practices (e.g. yoga). Participants were asked to rate the perceived usefulness of chosen CM by selecting one option on a 7- point Likert scale ranging from ‘strongly agree’ to ‘strongly disagree’, in response to the statement *“I found this therapy to be effective for my condition and/or helpful for me overall”* or “*I found this therapy to be effective in helping me manage my weight”.* We further trichomotised the responses into ‘strongly agree/agree (effective)’, ‘somewhat agree/neither agree nor disagree (neutral)’, and ‘somewhat disagree, disagree, strongly disagree (not effective)’.

Data on visits to conventional health practitioners (general practitioner/primary care physician, oncologist, allied health) over the past 12 months were also collected. Finally, we asked women about the perceived advantages and disadvantages of using CM for weight management and which CM they were willing to use for weight management if there was research evidence to demonstrate effectiveness. Women were given a selection of responses to choose from and could also provide free text answers for additional comment.

### Statistical analysis

IBM SPSS statistics package version 23 [11] and Stata statistical software [12] were used to analyse the data presented in this report. We used descriptive statistics to present the majority of the data, and Pearson’s chi-square to identify associations between weight gain and CM use, and advanced breast cancer (metastatic or inflammatory) and CM use. To explore differences in demographic characteristics between respondents who were from the BCNA Review and Survey group vs non BCNA respondents, we used Pearson’s chi-square, linear regression and independent samples t-tests.

## Results

### Survey response

Of the 1857 BCNA members, 283 (15%) responded to the survey. A further 26 women responded to the survey from other channels giving a total of 309 responses.

### Sample characteristics

Demographic and clinical characteristics of respondents are described in Table 1. The majority of women were Caucasian (92.5%, *n* = 285) with a mean age of 59.1 years (*SD* = 9.5, range 33–78, *n* = 298). Characteristics were similar across BCNA members and non-BCNA respondents except that there was a higher proportion of women in the non-BCNA group who were self-employed (23% vs 10%) and in the BCNA group who were retired (33% vs 23%), however this difference was not statistically significant on Pearson’s chi-square testing, *X*^*2*^ (7,*N* = 307) = 6.9912*, p =* 0.430. There was also no statistically significant correlation between age and type of employment apart from doing voluntary work (*p =* 0.05), and no difference in age between BCNA and non-BCNA respondents (*p* = 0.0759). The majority of women (82%, *n* = 252) had been diagnosed with non-metastatic BC. The mean time since diagnosis of BC was 8.2 years (SD 5.12, range 1–32 years) and mean age at diagnosis was 50.9 years (SD = 9.02, range 29–74). The majority of women were diagnosed either Ductal Carcinoma in Situ or localized breast cancer. There was no association between more advanced cancer and use of any complementary medicine, *X*^*2*^ (1, *N* = 289) = 2.1218, *p* = 0.145.

**Table 1:** Demographic and clinical characteristics of survey respondents

	Description	N (responses)	Percentage
State (*n* = 309)			
	Australian Capital Territory	14	4.53%
	New South Wales	91	29.45%
	Northern Territory	0	0.00%
	Queensland	48	15.54%
	South Australia	28	9.06%
	Tasmania	3	0.97%
	Victoria	95	30.74%
	Western Australia	30	9.71%
Education (n = 307)			
	High school- year 10	30	9.77%
	High school- year 12	35	11.40%
	Vocational College	55	17.91%
	Bachelor’s degree	90	29.32%
	Postgraduate degree	97	31.60%
Ethnicity (*n* = 308)			
	European/Anglo Saxon/Caucasian	285	92.53%
	Asian	5	1.62%
	Oceanic (incl. Australian and New Zealand first peoples, Polynesian and Micronesian)	13	4.22%
	North/South/Central American	2	0.65%
	Mixed ethnicity	2	0.65%
	Indian	1	0.33%
Employment (n = 308)			
	Employee	140	45.46%
	Self-employed	33	10.71%
	Home duties/caring for children or family	15	4.87%
	In education (going to school, university, etc.)	4	1.30%
	Doing voluntary work	10	3.25%
	Unable to work because of illness	6	1.95%
	Unable to work for other reasons	1	0.32%
	Retired	99	32.14%
Relationship Status (n = 309)			
	Single	39	12.62%
	Married/De Facto (living with partner)	230	74.43%
	In a relationship but not living with partner	7	2.27%
	Divorced/separated	24	7.77%
	Widowed	9	2.91%
Diagnoses (n = 308)			
	Ductal Carcinoma In Situ (DCIS)	33	10.71%
	Localised breast cancer	252	81.82%
	Metastatic breast cancer	14	4.55%
	Inflammatory breast cancer	2	0.65%
	Other including second primary	7	2.27%

### Weight change

Mean BMI at time of diagnosis was 26.23 kg/m^2^ (*n* = 270, SD 5.43) and at time of survey was 28.02 (*n* = 285, SD 5.88). Just under half of women (48.5%) were overweight or obese at time of diagnosis, but by the time of the survey this proportion had risen to 67.3%. This increase was most marked for women who were obese, from 17.04% at diagnosis to 31.93% at the time of the survey. The majority of respondents (63.70%) reported they had gained weight overall after diagnosis. Of the women who reported gaining weight overall and for whom we had complete weight data (*n* = 175), average weight gain was 9.07 kg.

### Complementary therapy use for any health condition

**Fig. 1:**
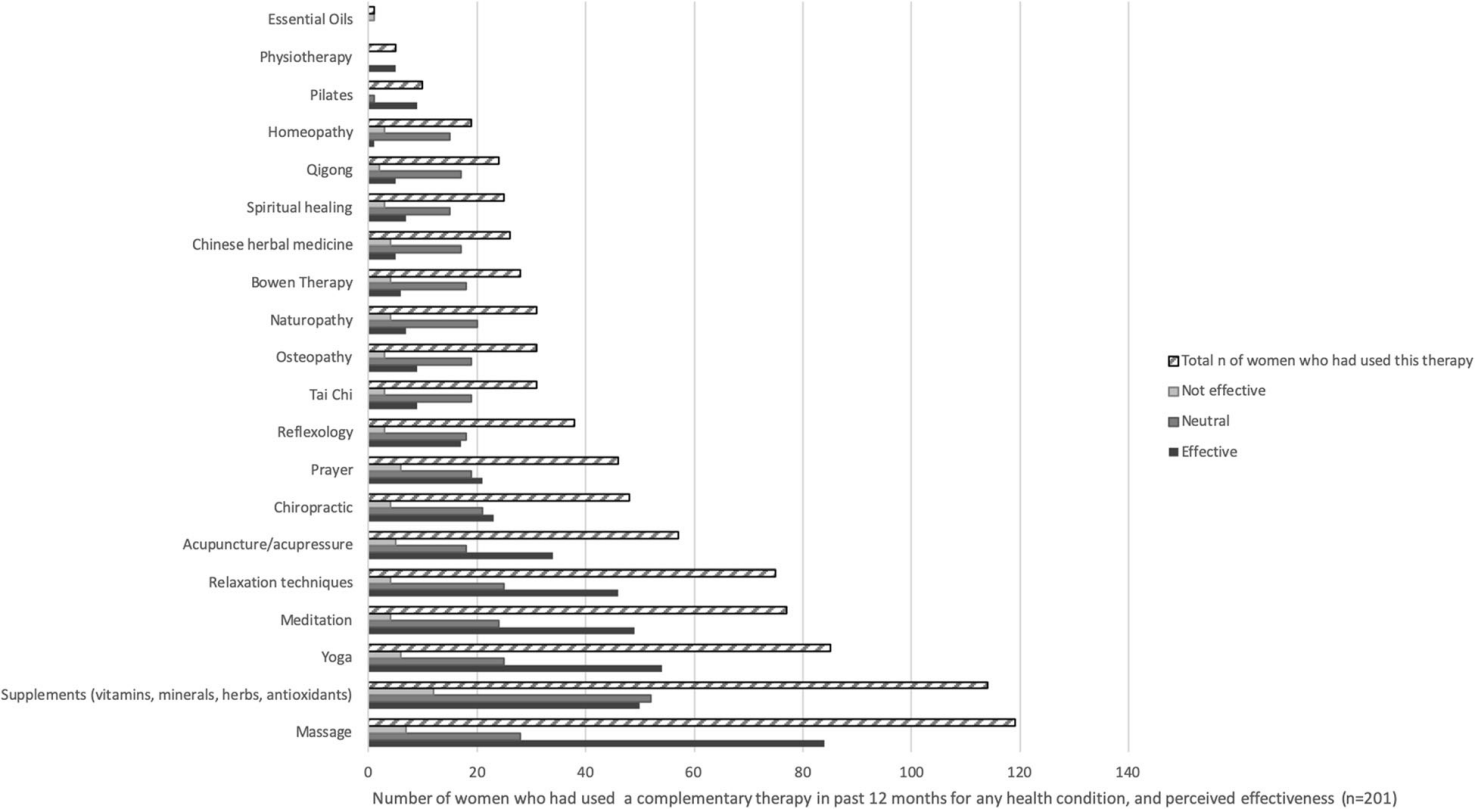
Complementary therapy use for any condition, and perceived effectiveness

Figure 1 describes reported CM use for any health condition and associated perceived effectiveness, and Table 2 describes reasons for CM use and information sources. About three quarters of women (201 or 73.4%) had used a CM for any health condition in the past 12 months. The top five CMs used to treat any health condition were relaxation techniques, yoga, meditation, prayer and massage. The top three reasons for CM use were to improve general physical wellbeing (80% of CM users), reduce stress/improve psychological wellbeing (62% of CM users), and to treat a condition unrelated to cancer (37% of CM users). The treatments that women found most effective were massage, yoga, meditation, acupuncture, and relaxation with more than 50% of women who had used these therapies strongly agreeing or agreeing that the treatments had been effective. Most women sought information on CM from friends and family, followed by complementary therapists, the internet, GPs, and specialists. There was no association between CM use and whether women had gained > 5% of weight overall or had maintained a stable weight, *X*^*2*^ (1, *N* = 277) = 0.2017, *p* = 0.653.

**Table 2:** Complementary therapy use: Reasons and information sources

	Description	Number	Percent
Reasons for CM use (*n* = 201)			
	Improve physical wellbeing	159	79.10%
	Stress management/improve psychological wellbeing	124	61.69%
	Improve a non-cancer related symptom or condition	75	37.31%
	Improve a side effect related to cancer treatment	51	25.37%
	Boost immune system	40	19.90%
	Prevent recurrence	39	19.40%
	Improve a cancer-related symptom	29	14.43%
Source of CM information (*n* = 184)			
	Friend/family	70	38.04%
	Complementary therapist	48	26.09%
	Internet	46	25.00%
	GP	43	23.37%
	Specialist	28	15.22%
	Media (TV, newspapers, magazines, radio)	23	12.50%
	Nurse	8	4.35%
	Social media	8	4.35%
	Pharmacist	2	1.09%

*CM*=Complementary medicine

### Complementary therapy use for weight loss

**Fig. 2:**
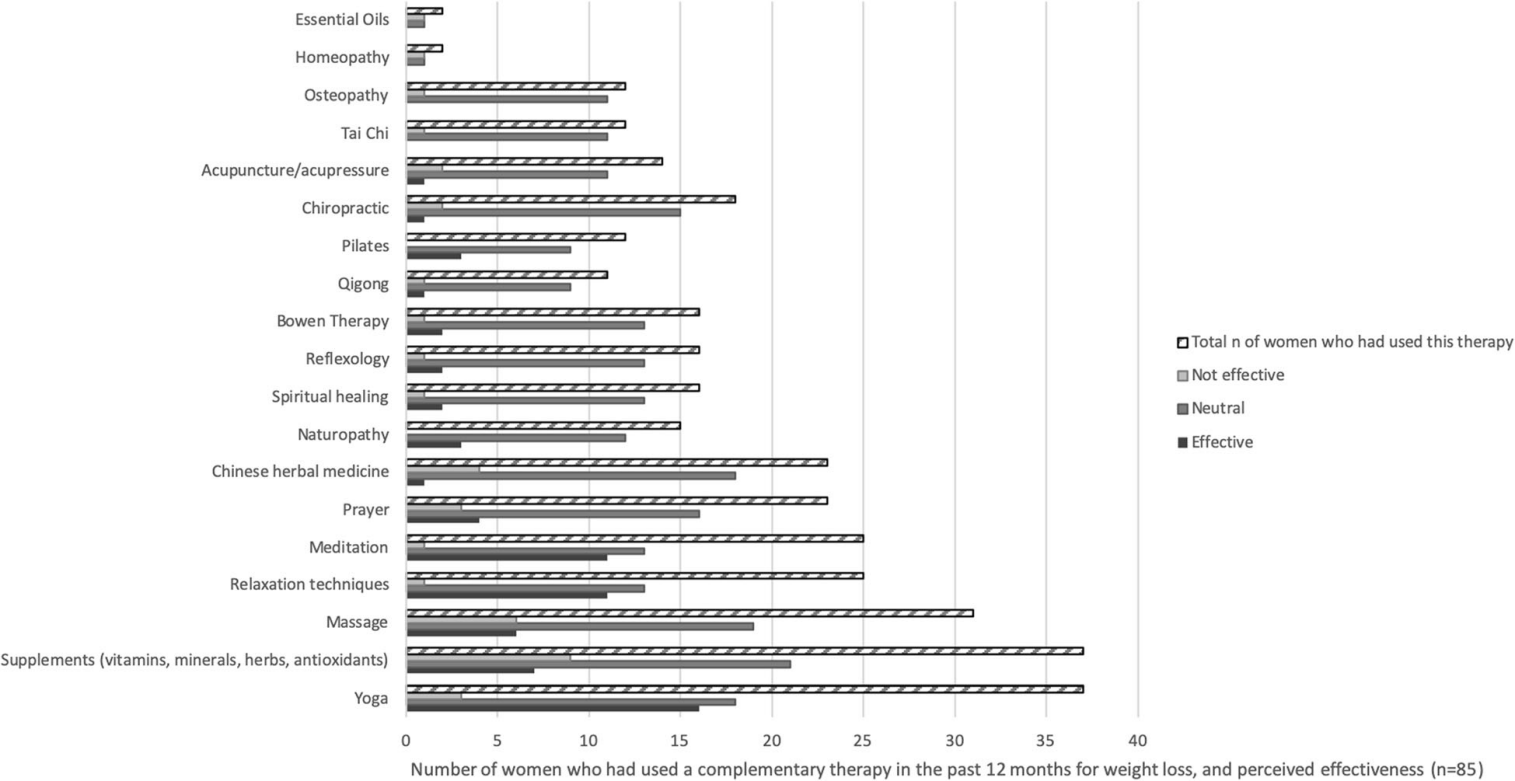
Complementary therapy use for weight loss, and perceived effectiveness

Figure 2 describes the number of women who had tried a CM for weight loss, and perceived effectiveness. A small number (*n* = 85, or 31% who completed the entire survey) of women had tried CM in the last 12 months for weight loss. The most popular therapies were supplements, yoga, relaxation techniques, massage and meditation. More than 40% of women who had tried yoga, meditation or pilates agreed or strongly agreed it had been helpful in relation to weight loss, however the majority of women felt neutral about the effectiveness of the therapies they had tried. Table 3 describes the perceived advantages and disadvantages of using CM for weight management. The most commonly selected perceived advantages of using CMs for weight loss were the ability to improve general wellbeing, relaxation, and being non-pharmacological. The major disadvantages reported were financial cost, finding a reliable practitioner, and lack of research for effectiveness.

**Table 3:** Perceived advantages and disadvantages of using complementary therapies for weight management

	Description	Number	Percent
Perceived advantages of CM use (*n* = 183)			
	Improves general wellbeing	139	75.96%
	Reduces stress/is relaxing	105	57.38%
	Not needing to use drugs/medication	101	55.19%
	Can treat other symptoms at the same time	64	34.97%
	Holistic approach	57	31.15%
	Non-surgical/not needing to have an operation	56	30.60%
	Agree with the philosophy behind the therapy	37	20.22%
	Already using it	18	9.84%
Perceived disadvantages of CM use (*n* = 214)			
	Financial cost	128	59.81%
	Lack of research to show it is effective	124	57.94%
	Finding a reliable practitioner	99	46.26%
	Interactions with medications	58	27.10%
	Cannot find reliable information	53	24.77%
	Time commitment	37	17.29%
	Side effects of treatment	14	6.54%
	My GP/specialist does not support its use	13	6.07%
	Don’t work/don’t trust	3	1.40%
	Unsure	2	0.93%
	Motivation to continue	1	0.47%
	Too many treatments already	1	0.47%
	Goes against my beliefs	1	0.47%

*GP*=General Practitioner/family physician/primary care physician

**Fig. 3:**
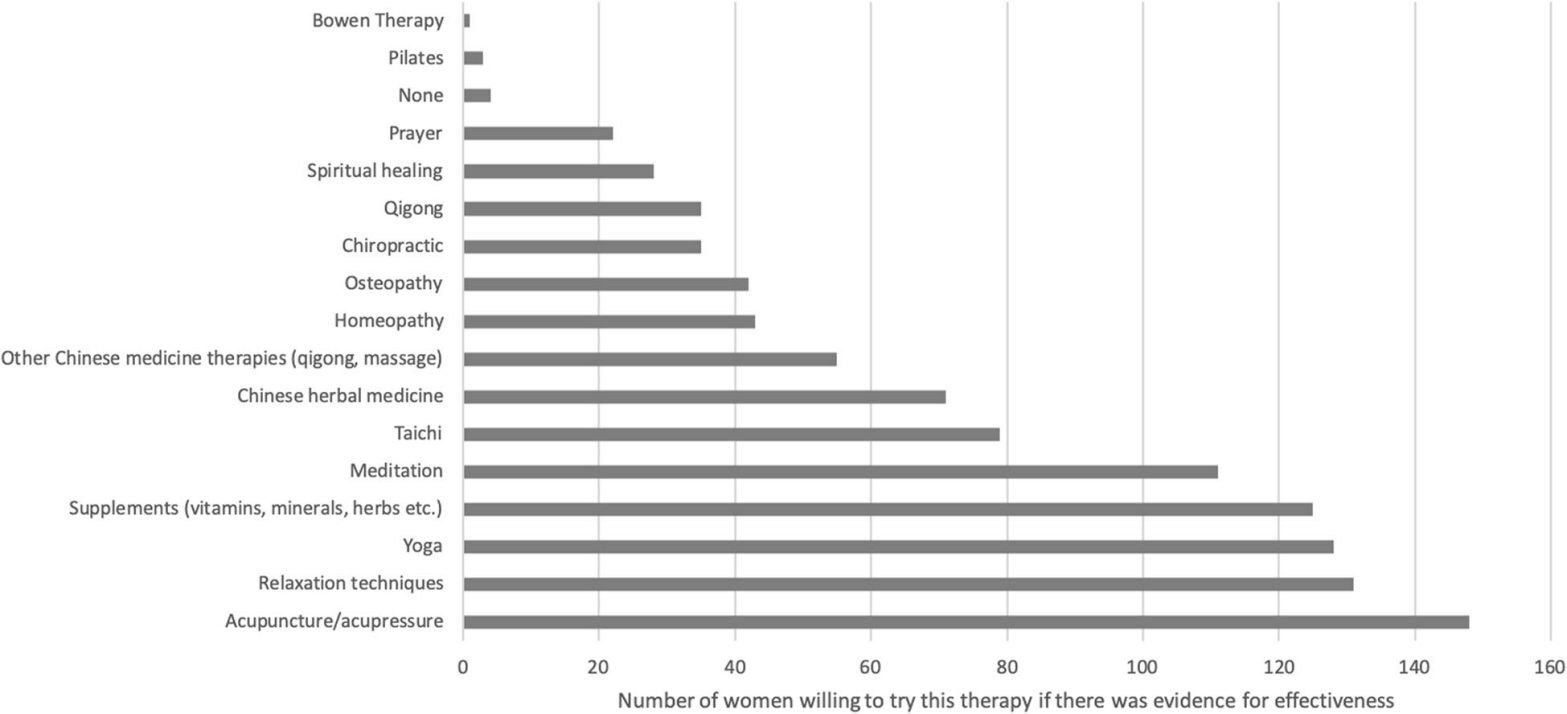
Complementary therapies that respondents would be willing to try if effective for weight loss

When asked which therapies they would try if there was research to demonstrate effectiveness for weight loss, almost half of women indicated they would be willing to try acupuncture/acupressure, followed by relaxation techniques, yoga, supplements, and meditation (Fig. 3). Overall, around three quarters of respondents (237 women) would be willing to try a CM for weight loss if there was demonstrated scientific evidence for its effectiveness.

## Discussion

In our survey, we found high rates of CM use for any condition, but lower rates of use for weight management, with limited perceived effectiveness of the therapies that had been tried. Women in our survey cited barriers to use of CM for weight management after BC including lack of research for effectiveness. However, CM was seen to be advantageous in improving general wellbeing, providing relaxation, and being non-pharmacological. Three quarters of women would consider a CM if there was evidence for effectiveness (particularly, acupuncture/acupressure, relaxation techniques, yoga, supplements, and meditation).

Almost three quarters of women in our sample had used a CM in the preceding 12 months. This is consistent with evidence from a recent systematic review reporting prevalence of up to 87% in Australia [3]. CM users in our sample cited reasons for use such as improving general physical wellbeing, reducing stress/improving psychological wellbeing and treating conditions unrelated to cancer. Similarly, in another survey, BC survivors use CMs to “help healing, to promote emotional health, and to cure cancer” [3]. The most recent survey conducted in Australia on CM use in BC survivors reported women believed that CMs improved their wellbeing, boosted their immune system, reduced side effects of treatments, reduced symptoms of cancer, treated the cancer, and prevented recurrence [13]. Cancer patients mostly report using CM in an adjunctive manner, e.g. to improve overall general health and wellbeing [14] or to minimize adverse effects from conventional treatment and to prevent further illness [15]. Collectively, these data suggest that women with BC seek a range of therapeutic options to optimize all aspects of their health and wellbeing in a holistic manner, particularly to improve psychological wellbeing.

In our sample, the most commonly used CMs for any reason were nutraceutical supplements, massage, meditation and yoga which is consistent with what is reported in the literature [3]. Some studies specifically reported whole medical systems such as naturopathic or traditional Chinese medicine most commonly used by BC patients [16, 17]. Of interest, although nutraceutical supplements were the most commonly used therapy, about half of women perceived their effectiveness to be neutral. The therapies with the highest perceived effectiveness included massage, acupuncture/acupressure, relaxation techniques and yoga.

A smaller proportion (31%) of women had used CM for weight management. In non-cancer populations, studies suggest that up to 70% of people with obesity use CM [18, 19] particularly if they are female [18]. People with metabolic syndrome are also higher users of CM [19] compared to people without metabolic syndrome. However, it is unclear if the high use of CM in people without obesity indicates use specifically for weight management, or if CM are used for other reasons [18, 19]. We could not find any literature describing the prevalence of use of CM for weight management in BC survivors. The therapies were mostly perceived as neutral in terms of effectiveness, with the exception of yoga and meditation, which greater than 40% of our sample thought were effective treatments.

One of the most commonly cited barriers to using CM in our study was the perceived lack of evidence for effectiveness. Similarly, in a qualitative study, the most common reason given for deciding not to use CM amongst cancer survivors was a lack of meaningful information regarding safety and efficacy [15]. However, around three quarters of women in our sample indicated that they would try a CM to assist with weight management should there be sufficient evidence demonstrating effectiveness. The most commonly cited CM that would be chosen in this situation was acupuncture/acupressure, with around half of women willing to trial these modalities, followed by relaxation techniques, yoga, supplements, and meditation. Indeed, acupuncture shows promise in the treatment of obesity and overweight in general populations. A recent meta-analysis reported that acupuncture, in particular auricular acupuncture and electro-acupuncture, was more efficacious than sham acupuncture for reducing BMI (MD − 0.47 kg/m^2^) as well as body fat mass (MD − 0.66 kg), waist circumference (MD − 2.02 cm) and hip circumference (MD − 2.74 cm) but not for reducing body weight overall [20]. Mechanistic studies have suggested multiple responses to acupuncture including appetite suppression [21, 22], modulation of leptin and ghrelin [23–25] and improved insulin sensitivity [26–31]. Further, acupuncture may alleviate co-morbid anxiety symptoms in people with obesity [23, 32, 33]. Given that acupuncture is a relatively safe treatment [34–36] and may have additional benefits in women with BC including relief of chemotherapy-induced peripheral neuropathy [37], aromatase-inhibitor induced arthralgia [38, 39], menopausal symptoms [40] and lymphoedema [41], it may represent a useful adjunctive therapy that can assist women in managing a number of bothersome symptoms and manage weight. To the best of our knowledge, there are no studies examining the effectiveness of acupuncture for weight loss in women with BC, and such research appears warranted.

Women in our sample were also willing to trial meditation, yoga and nutritional supplements for weight management. In non-BC populations, limited evidence suggests that mindfulness meditation may help people improve eating behaviours (such as reducing the amount of emotional eating), increase physical activity, and reduce anxiety and stress [42–47] while two pilot studies using mindfulness-based techniques for weight management in women with BC have reported promising findings for weight loss and eating behaviors [48, 49]. Again in non-BC populations, yoga may be effective for reduction of BMI compared with usual care (SMD -0.99) [50], while a pilot trial in women with BC reported a reduction in waist circumference of 3.1 cm and improvements in quality of life [51]. A range of nutraceutical supplements may have modest effects on weight [52–55]. Given the interest in using these complementary modalities to assist weight loss, and the potential for additional benefits such as for mental health, further clinical research into the effectiveness and efficacy of these low risk mind-body interventions as an adjunct to lifestyle interventions is required.

This survey has some strengths and limitations. We achieved a higher than expected response rate from the BCNA Review and Survey Group, which is typically 10% (email communication with BCNA Review and Survey Group,3 Oct 2017). We were also able to recruit across Australia, with the percentage of respondents from each Australian State and Territory in our study being similar national averages on BC incidence sourced from the Australian Institute of Health and Welfare cancer data [56]. However, the majority of women from the BCNA Review and Survey Group did not respond, and the demographics of this group are also unclear. Also, the total numbers of women who used CM for weight management were small. These factors limit the validity of our findings.

## Conclusion

We found evidence for high prevalence of use of CMs in a sample of BC survivors to improve wellbeing and relieve symptoms. The use of CM for weight loss is more limited, most probably due to concerns over lack of evidence for efficacy and other barriers such as financial cost. However, three quarters of women in our sample would be willing to try CMs such as acupuncture/acupressure, meditation, yoga, and nutritional supplements should there be relevant supporting evidence for efficacy for weight management. Our findings should be interpreted cautiously given the small numbers of women who were using CM for weight loss, and the low response rate of our survey. Given the burden of weight after BC, further research into these modalities is warranted.

## Data Availability

The datasets used and/or analysed during the current study are available from the corresponding author on reasonable request.

## Appendix

### Specific demographic, medical, menopausal and lymphoedema details requested in the survey

#### Demographic characteristics

State of residence, highest level of education, ethnicity, employment status, relationship status, current age and age at diagnosis were included to describe the characteristics of women.

#### Medical details

Women were asked about their diagnosis, treatments received including treatments received to the axilla, the number of lymph nodes removed, whether they had a reconstruction, use of hormonal treatments, menopausal state (at diagnosis and current), presence of other medical conditions and symptoms such as hot flushes and the presence and severity of lymphoedema.

Women were asked to describe the type of breast cancer they were diagnosed with as either “ductal cancer in-situ (DCIS)”, “localised stage breast cancer (where your breast cancer is contained within your breast and/or lymph nodes), “metastatic breast cancer (breast cancer that has spread beyond the breast tissue and lymph nodes to distant parts of the body, such as the bones, liver and lungs; also called advanced, secondary or stage four)“ or “inflammatory breast cancer“. For convenience, inflammatory breast cancer and metastatic breast cancer were then combined and referred to as advanced breast cancer. Women were also asked to indicate the treatments they received such as “Lumpectomy alone”, “Lumpectomy and radiation”, “mastectomy alone”, “mastectomy and radiation”, “removal of lymph nodes”, “chemotherapy”, “hormonal therapy”, “targeted therapy (Herceptin)”, and “other”. As chemotherapy is invariably not provided to women with DCIS, we recoded the diagnosis as “localised” if a woman indicated that she had received chemotherapy.

Menopausal state at the time of diagnosis was assessed as either “Premenopausal (regular periods with no menopausal symptoms such as hot flushes)”, “Perimenopausal/in the menopausal transition (no periods for at least 2 months, plus hot flushes)”, “Postmenopausal (no periods for at least 12 months)” or “Previous surgical menopausal (both ovaries or uterus/womb had been removed).” Participants who indicated they were premenopausal or perimenopausal at the time of diagnosis were asked if they were having periods before breast cancer treatment and to describe what has happened to their periods now; “they have stopped”, “they stopped and then started again”, “they have become more irregular”, “no change” or “other”.

Lymphoedema severity was defined as either “no problem (no noticeable swelling)”, “mild (soft swelling that is not obvious to others and comes and goes)”, “moderate (swelling with occasional hardness in some areas that is obvious to others and is always present)”, “severe (profuse swelling with thickened skin, constant hardness, and a very large, heavy arm that is extremely obvious to others and is always present) as described elsewhere [7].”

#### Lifestyle habits

Women were asked if they had tried the following specific diets in the previous 12 months: Atkins diet (low carbohydrate), 5:2 diet (eat what you want 5 days a week, send your body into starvation mode for 2 days), Paleolithic diet, Dukan diet (High-protein, low-carb), Vegetarian diet, Vegan diet, Weight Watchers diet, Raw food diet, Ultra low-fat diet, Zone diet, Cambridge diet (very low calories), South Beach diet (low-GI), Other. They were asked if they ate at least the recommended serves of fruit and vegetables a day (2 fruit, five vegetable) with answer options of Yes/No. Self-perceived diet quality was assessed as Excellent/Very Good/Good/Fair/Poor. Smoking was assessed as current cigarrete use (Never smoked/Ex smoker/Recently quit ex smoker (smoked in the last 3 months)/Current smoker) and current smokers were asked to indicate the number of cigarettes they smoked each day. Alcohol intake was assessed as Non drinker/1–7 standard drinks a week/8–14 standard drinks a week/> 14 standard drinks a week) and a guide to standard drink sizes was provided. The validated Weight Self Efficacy Scale (WEL-SF) [3] was used to evaluate how confident women now felt about being able to successfully resist the desire to overeat in eight different situations on an 11-point Likert scale from 0 (not confident at all) to 10 (very confident). We further dichotomised the responses into “Not confident” (0–4) and “Confident” (5–10). Physical activity levels were calculated according to the number of 20-min sessions of less vigorous exercise or more vigorous exercise a week, given a weighting and described in terms of MET (metabolic cost) minutes where MET minutes less than 80 were coded as no physical activity, 80 to 400 as low, 400 to 560 as moderate and more than 560 as high. A value of 4 METs was given to moderate physical activity and 7.5 to vigorous physical activity [26].

#### Weight management

Experiences with a range of weight loss interventions (Exercise, Diet – various: Intermittent fasting, etc. (please specify), Meal replacements e.g. shakes, Medication, Weight loss supplements/products, Surgery (please specify), Online program e.g. 12 week Body Transformation, Social support, Weight loss program e.g. Jenny Craig, Psychological treatments such as CBT (Cognitive Behavioural Therapy) and the perceived effectiveness of the interventions on was described using a five-point Likert scale from 1 (not at all effective) to 5 (very effective). The responses were further dichotomized into 1 to 2 (not effective) and 3 to 5 (effective). Women were also asked about perceived barriers and facilitators to successful weight loss and weight maintenance, and what they believed should be research priorities in this area.

## Abbreviations

BC: Breast Cancer
BCNA: Breast Cancer Network Australia
BMI: Body Mass Index
CM: Complementary Medicine
DCIS: Ductal Carcinoma In Situ
GP: General Practitioner
